# Prenatal Diagnosis and Perinatal Management of Acrania Progressing to Anencephaly

**DOI:** 10.7759/cureus.117228

**Published:** 2026-09-30

**Authors:** Cesar Alas-Pineda, Oswaldo Montes Bardales, Anahi Pacheco, Sendy Ruiz, Elijah J Remis, Simmons Gough

**Affiliations:** 1 Quantitative Biomedical Science Program, Dartmouth College, Hanover, USA; 2 Faculty of Medicine and Surgery, Catholic University of Honduras, San Pedro Sula, HND; 3 Geisel School of Medicine, Dartmouth College, Hanover, USA; 4 Department of Radiology, Hospital Civil de Guadalajara Fray Antonio Alcalde, Guadalajara, MEX

**Keywords:** acrania, mickey mouse sign, neural tube defect, obstetric ultrasound, prenatal diagnosis

## Abstract

Acrania is a severe congenital malformation characterized by partial or complete absence of the cranial vault. We report the case of a 24-year-old woman (G2P1) who presented at 34+1 weeks of gestation with labor contractions. Prenatal care began at 7 weeks, and ultrasound examinations at 12 and 25 weeks confirmed fetal acrania with persistent breech presentation. Given the advanced gestational age, persistent breech presentation, and multidisciplinary counseling, cesarean delivery was performed. A 3,100g neonate was delivered with anomalies consistent with the prenatal diagnosis and died 10 hours later while receiving comfort-focused palliative care. The mother recovered uneventfully and was discharged in stable condition. This case highlights the clinical importance of early prenatal recognition of acrania and individualized perinatal planning, particularly when the diagnosis persists into advanced gestation. Early diagnosis facilitates informed counseling and coordinated obstetric and neonatal management, including appropriate preparation for palliative care.

## Introduction

Fetal acrania is a severe congenital anomaly classified among neural tube defects (NTDs), and it is characterized by partial or complete absence of the cranial vault resulting from disruption of mesenchymal migration during early embryogenesis. Acrania is considered the initial stage of the acrania-exencephaly-anencephaly sequence. In the absence of the cranial vault, the exposed cerebral tissue progressively degenerates due to mechanical and chemical injury from the amniotic environment, leading to exencephaly and, with further loss, anencephaly, a lethal malformation incompatible with life characterized by severe absence of cerebral tissue [1-3]. 

The incidence of acrania is estimated at 3.68 to 5.4 cases per 10,000 live births, whereas anencephaly occurs in approximately 1 in 1,000 births. These anomalies are typically diagnosed via ultrasonography at the end of the first trimester or early in the second trimester. A characteristic sonographic feature is visualization of cerebral tissue floating in the amniotic fluid above the cranial base, described as the “Mickey Mouse sign” [4]. Differentiation between acrania and extensive encephalocele may be clinically challenging due to overlapping imaging findings [1]. 

The prognosis of the acrania-exencephaly-anencephaly sequence is uniformly poor, and most affected fetuses do not survive birth. A recent study reported that 58% cases resulted in intrauterine fetal demise, while most live-born neonates died within the first 24 hours of life. In rare cases, survival of up to one week has been documented. Given the absence of curative treatment and the lethal prognosis, termination of pregnancy may be considered. For pregnancies that continue, management should include comprehensive prenatal follow-up and monitoring for complications such as polyhydramnios, which occurs in 25-50% of cases [1,4]. 

To reduce the risk of recurrence of NTDs, preconception supplementation with 4 mg of folic acid daily is recommended and has been shown to significantly decrease their incidence [1,2]. 

Although acrania can be diagnosed during the first trimester, the literature provides limited description of perinatal management when the diagnosis persists into advanced gestation, particularly when complicated by persistent breech presentation and the need for coordinated obstetric and neonatal planning. This case is important because acrania was diagnosed early in pregnancy and subsequently progressed toward anencephaly; however, structured multidisciplinary perinatal planning became relevant only when the patient presented in the third trimester with persistent breech presentation and preterm labor. We report on this case to emphasize the importance of early multidisciplinary counseling and individualized obstetric planning following prenatal diagnosis of a lethal fetal anomaly. 

## Case presentation

We present the case of a 24-year-old woman (Gravida 2, Para 1, Abortions 0, Living Children 1, Fetal Deaths 0, Cesarean Sections 0) at 34 weeks and 1 day of gestation based on the last menstrual period. She was admitted with severe colicky lower back pain radiating to the hypogastric region, accompanied by abdominal tightening and regular uterine contractions beginning 12 hours prior to presentation. 

The patient reported initiating prenatal care at 7 weeks of gestation and adherence to folic acid and iron supplementation. At 12 weeks, ultrasonography detected a congenital malformation consistent with acrania, characterized by complete absence of cranial ossification and exposed brain tissue without clear evidence of degeneration (Figure 1). 

**Figure 1:**
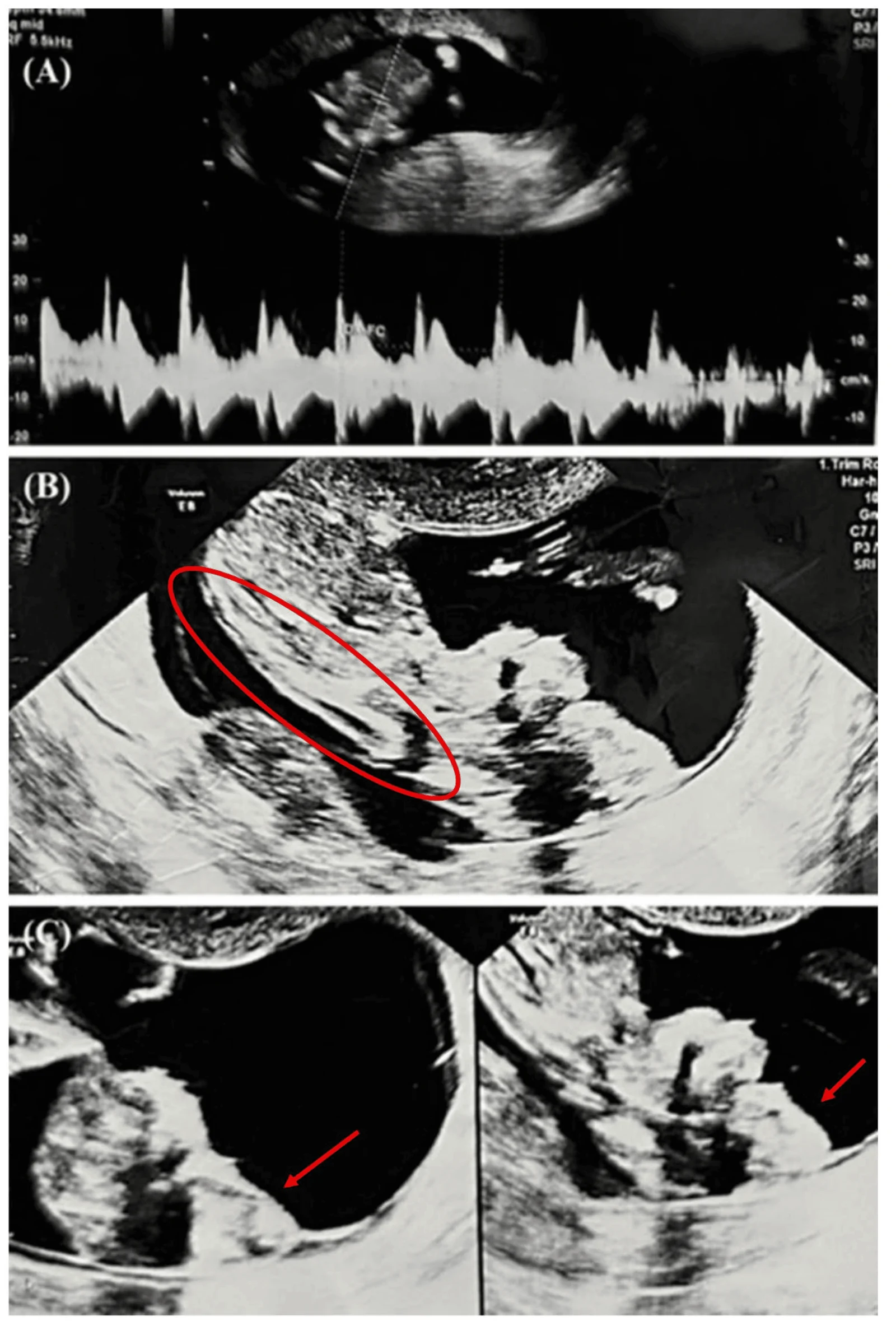
Suprapubic fetal ultrasound performed at 12 weeks of gestation (dating by last menstrual period). Cardiac evaluation (A): Four-chamber cardiac view demonstrates normal anatomy with a confirmed fetal heart rate of 160 beats per minute. Spinal evaluation (B): Longitudinal spinal view showing continuous vertebral column without apparent neural tube defects. Cranial evaluation (C): Transverse and longitudinal views of the fetal calvarium demonstrating absence of cranial ossification with exposed, disorganized cerebral tissue protruding into the amniotic cavity, without evidence of tissue loss or degeneration, consistent with fetal acrania.

A follow-up ultrasound at 25 weeks confirmed breech presentation and the expected progression toward anencephaly, with marked resorption of exposed neural tissue (Figure 2). 

**Figure 2:**
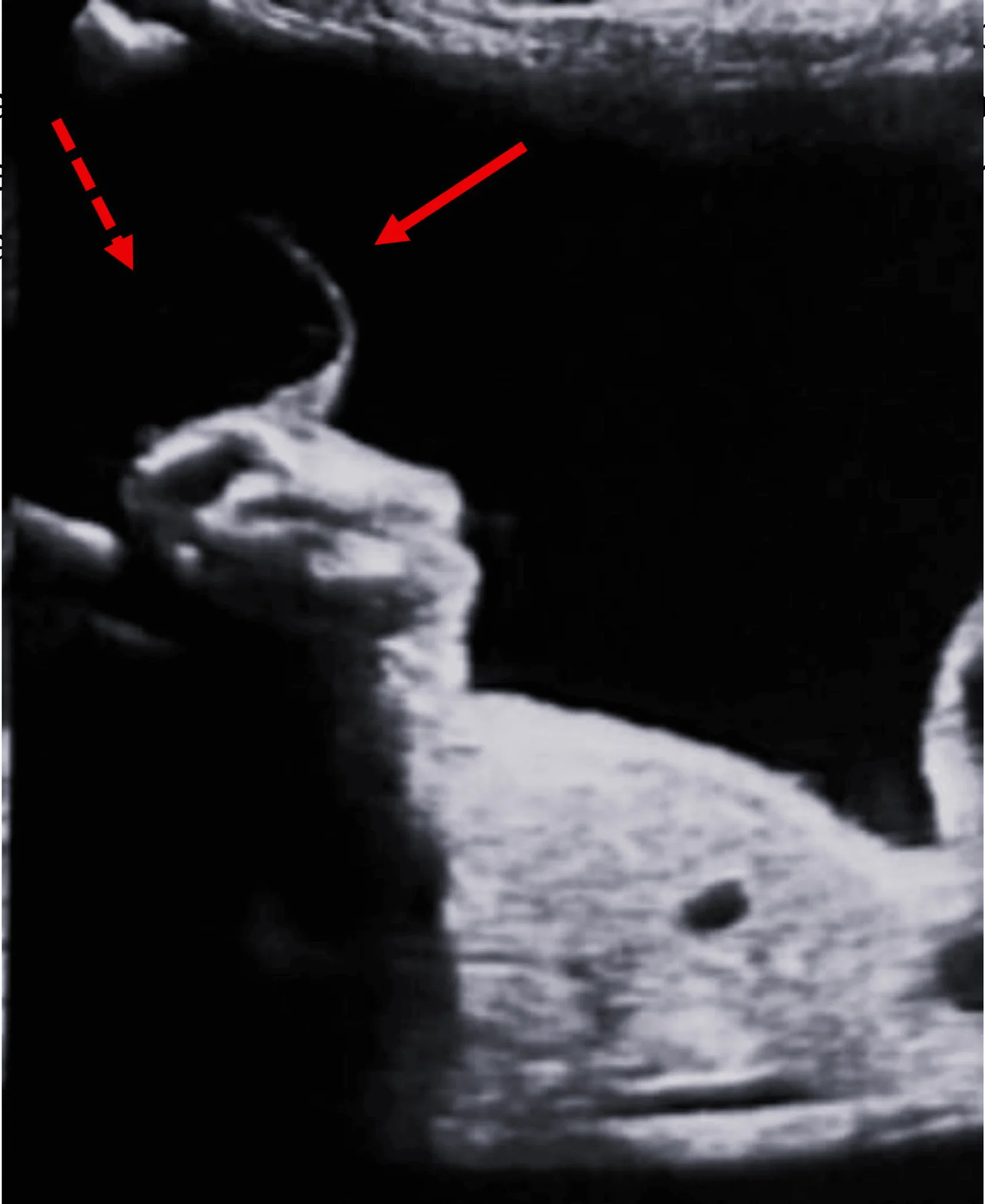
Suprapubic fetal ultrasound performed at 25 weeks of gestation. Persistent absence of cranial bone ossification is observed with continued absence of encephalic tissue within the cranial vault and replacement by fluid-filled structures.

The chronological sequence of clinical events is summarized in Table 1. 

**Table 1:** Timeline of clinical events

Gestational age	Clinical event
7 weeks	Prenatal care initiated
12 weeks	Fetal acrania diagnosed by ultrasonography
25 weeks	Progression toward anencephaly confirmed
34+1 weeks	Admission for preterm uterine contractions
34+2 weeks	Cesarean delivery
Postpartum	Neonatal death at 10 hours; uncomplicated maternal recovery

The patient had no significant personal or family medical history of neural tube defects or genetic disorders. Her first pregnancy resulted in the birth of an infant with a cleft palate and no additional congenital anomalies or underlying conditions. She denied exposure to known teratogenic medications and reported no use of tobacco, alcohol, or illicit drugs. Prenatal follow-up was regular throughout the pregnancy. 

On admission, physical examination revealed regular uterine contractions occurring three to four times every 10 minutes, moderate lumbar pain, and cervical changes suggestive of early labor. No other clinically significant physical abnormalities were noted. Ultrasonographic evaluation confirmed the absence of the cranial vault and the presence of disorganized, exposed neural tissue, consistent with acrania progressing to anencephaly. 

The decision for cesarean delivery reflected a combination of obstetric and contextual factors rather than the fetal anomaly itself, given the uniformly lethal prognosis. The primary obstetric indication was persistent breech presentation in the setting of active preterm labor. This combination is associated with an increased risk of maternal and intrapartum complications with vaginal delivery. The fetal anomaly was not itself an indication for cesarean delivery and is generally preferred in confirmed lethal anomalies to minimize maternal surgical risk. In our case, the delivery route was determined primarily by the obstetric considerations. Maternal preference, after counseling on the risks and expected outcomes of both delivery routes, and institutional resource availability for managing preterm breech labor, were also considered in the final multidisciplinary decision. Cesarean delivery was performed on the second day of hospitalization without major complications. A female neonate weighing 3,100 g was delivered with findings consistent with the prenatal diagnosis (Figure 3). 

**Figure 3:**
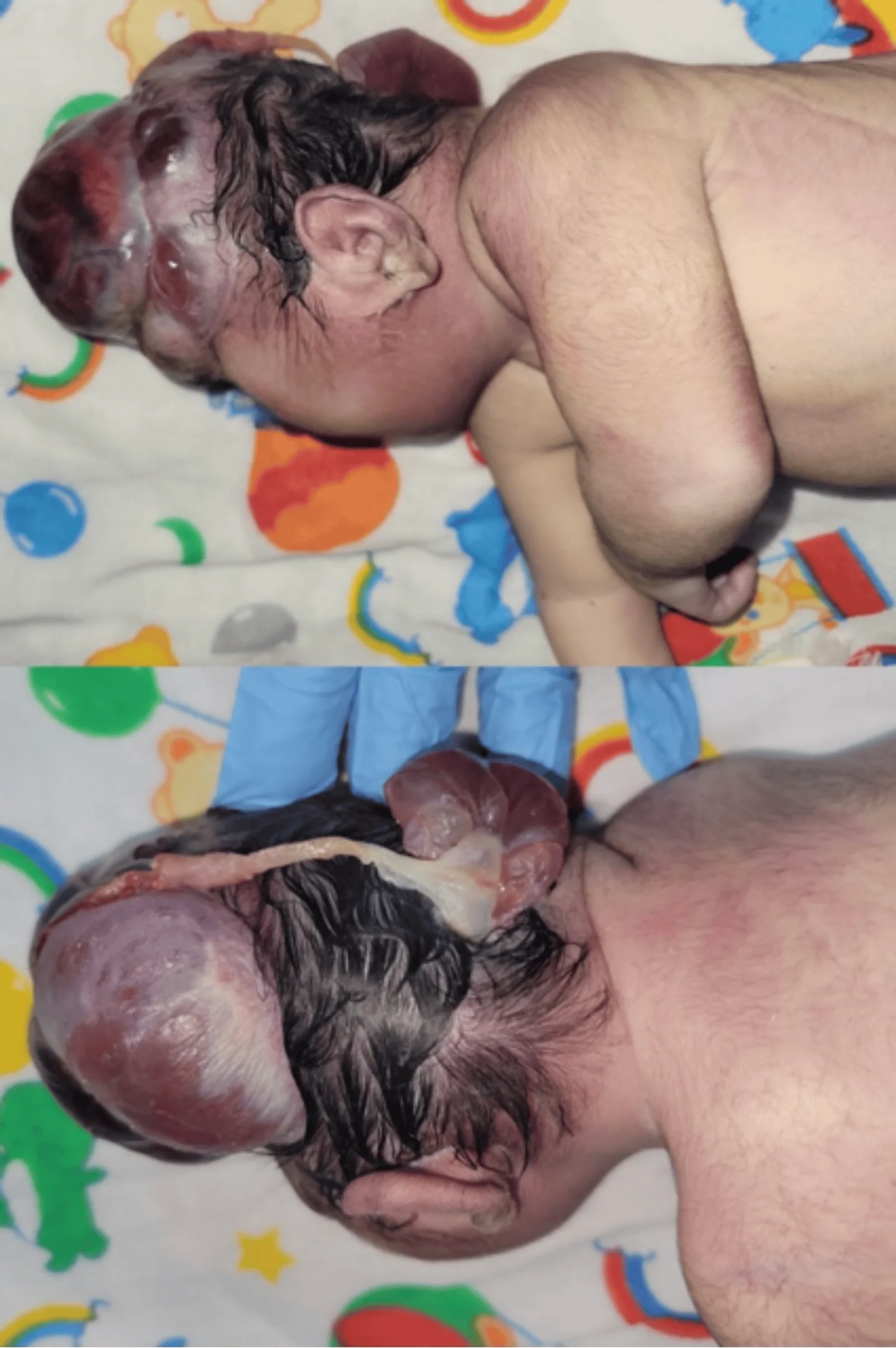
Newborn with severe acrania and anencephaly at birth. Image showing complete absence of the cranial vault and calvarial soft tissue, with the absence of cerebral tissue within the cranial cavity; residual structures are limited to a fluid-filled membranous covering, consistent with the predicted progression from acrania (Figure 1) to anencephaly on the ultrasonography.

Comfort-oriented palliative care was provided, and the neonate died 10 hours after birth. The mother’s postoperative course was uncomplicated, and she was discharged three days later. Prior to discharge, postpartum counseling and emotional support were provided. At the 6-week postpartum follow-up, the patient demonstrated satisfactory physical recovery with no unexpected adverse events. 

## Discussion

Acrania is among the most lethal congenital anomalies, with an estimated incidence of 3.68 to 5.4 per 10,000 live births. However, some studies report higher incidence rates of approximately 1:1,000 pregnancies [4]. The absence of the cranial vault results in direct exposure of cerebral tissue, which progressively degenerates due to fetal movement and chemical injury from amniotic fluid, leading to destruction and eventual absence of cerebral tissue, culminating in anencephaly. The combined acrania-anencephaly spectrum remains rare, with an estimated incidence of approximately 10 per 10,000 births [5]. 

A diagnosis of acrania is typically achievable between 11 and 13 weeks of gestation [6]. Ultrasonography remains the primary diagnostic modality, while fetal MRI should be reserved for cases of diagnostic uncertainty or suspicion of additional structural defects [7,8]. In resource-limited settings, ultrasound provides an accessible and cost-effective tool for early detection. 

We strongly emphasize the importance of first-trimester ultrasonography, as it allows early detection of congenital malformations and facilitates timely decision-making in perinatal planning [9]. Although ultrasonography is operator-dependent, in developing countries such as Honduras, where access to advanced imaging modalities like fetal MRI is limited due to economic constraints and a shortage of trained personnel, ultrasound has become a cost-effective and accessible diagnostic tool. 

Prenatal screening can complement early ultrasonographic evaluation in pregnancies at risk for fetal structural anomalies. Maternal serum alpha-fetoprotein (AFP), typically obtained during the second trimester, may be elevated in pregnancies affected by open neural tube defects because fetal AFP can enter the amniotic fluid as a result of neural tube exposure, thereby providing an additional screening marker [10]. An abnormal AFP result may thus prompt targeted ultrasonographic evaluation. Although AFP screening would not replace first-trimester ultrasonography for the detection of acrania, it may provide an additional opportunity to identify pregnancies requiring further evaluation. In the present case, the patient's previous child had an isolated cleft palate without other reported congenital anomalies. Therefore, this history alone did not establish a recognized risk factor for neural tube defects. Nevertheless, a history of a previous pregnancy affected by a congenital anomaly may support careful review of prenatal screening and detailed anatomic assessment in subsequent pregnancies. Where available, high-resolution ultrasonography remains central to the prenatal diagnosis of acrania, while genetic or molecular testing may be considered when additional anomalies or clinical findings raise suspicion for an underlying genetic condition.

Raising awareness among pregnant populations regarding early prenatal screening is particularly critical in the context of severe neurological congenital malformations. In the present case, given the maternal history of a previous child with a cleft palate, this case further highlights the importance of early ultrasound evaluation [11]. The clinical course in this case can be contextualized against other reported presentations of the acrania-exencephaly sequence. Jagtap et al. described a fetus diagnosed at approximately 15 weeks of gestation, with the pregnancy managed shortly after diagnosis [12]. Munihire et al. reported a case in which the anomaly was not apparent on an early scan and was only identified at birth, following delivery at 39 weeks [13]. Although the diagnosis in this case was made appropriately early (at 12 weeks), this case illustrates that early sonographic detection alone is insufficient without prompt initiation of structured multidisciplinary counseling and perinatal planning. The interval between diagnosis and the eventual preterm presentation represented a missed opportunity to establish an individualized delivery and palliative care plan before the patient arrived in active labor. A limitation of this report is the absence of fetal magnetic resonance imaging and genetic testing, which were not available in the clinical setting. This case report was prepared following the CARE guidelines [14]. 

## Conclusions

In conclusion, this case demonstrates that early prenatal diagnosis of a lethal fetal anomaly does not by itself ensure adequate perinatal management. Delayed or incomplete multidisciplinary counseling and perinatal planning can leave patients without a structured management plan by the time of delivery. This case demonstrates how timely diagnosis (initiated at 12 weeks and confirmed at 25 weeks) enables optimal prenatal counseling and multidisciplinary delivery planning before the onset of preterm labor. Such structured coordination is particularly vital in low-resource settings where access to specialized prenatal and genetic services may be limited. 
